# Prospective Evaluation of Three-Fraction SBRT with 4D Transperineal Ultrasound Intrafraction Motion Monitoring for Localized Prostate Cancer

**DOI:** 10.3390/cancers18182954

**Published:** 2026-09-13

**Authors:** Ilenia Manno, Matteo Mombelli, Valeria Faccenda, Sara Saufi, Giulia Rossano, Lorenzo De Sanctis, Federica Ferrario, Denis Panizza, Stefano Arcangeli

**Affiliations:** 1 School of Medicine and Surgery, University of Milano-Bicocca, 20126 Milan, Italy; i.manno2@campus.unimib.it (I.M.); m.mombelli8@campus.unimib.it (M.M.); valeria.faccenda@irccs-sangerardo.it (V.F.); s.saufi@campus.unimib.it (S.S.); g.rossano1@campus.unimib.it (G.R.); l.desanctis2@campus.unimib.it (L.D.S.); denis.panizza@unimib.it (D.P.); 2 Medical Physics, Fondazione IRCCS San Gerardo dei Tintori, 20900 Monza, Italy; 3 Radiation Oncology, Fondazione IRCCS San Gerardo dei Tintori, 20900 Monza, Italy; federica.ferrario@irccs-sangerardo.it

**Keywords:** prostate cancer, SBRT, quality of life, intrafraction motion

## Abstract

**Background/Objectives**: We aimed to prospectively evaluate the safety, patient-reported quality of life (QoL), and early oncological outcomes of a three-fraction SBRT regimen with real-time transperineal ultrasound guidance in localized prostate cancer. **Methods**: This prospective single-centre study enrolled patients with localized prostate cancer of all risk groups treated with SBRT (30 Gy in three fractions) between November 2023 and October 2025. Treatment was delivered using VMAT with CBCT-based setup verification and continuous real-time transperineal ultrasound for intrafraction motion monitoring. The primary endpoint was the incidence of acute and late grade ≥ 2 genitourinary (GU) and gastrointestinal (GI) adverse events (AEs) according to CTCAE v5.0. Secondary endpoints included patient-reported QoL (IPSS, EPIC-CP, IIEF-5), biochemical recurrence-free survival (BRFS), PSA kinetics, and factors associated with treatment-related AEs. **Results**: Seventy-two patients (median age, 78 years; range, 56–87) were enrolled, with a median follow-up of 16 months (range, 6–27). Acute grade ≥ 2 GU and GI AEs occurred in 34.7% and 8.3% of patients, respectively; late grade ≥ 2 GU and GI AEs occurred in 13.9% and 4.2%. One acute and one late grade 3 GU event were observed, with no grade 4–5 AEs. Although several QoL scores changed significantly over time, none exceeded the threshold for clinically meaningful deterioration. Higher baseline IPSS was associated with acute grade ≥ 2 GU AEs (aOR = 1.12, 95% CI: 1.00–1.26; *p* = 0.045). Median PSA declined from 7.90 to 0.44 ng/mL, and estimated BRFS was 98.2% at 12 months and 96.1% at 24 months. **Conclusions**: Three-fraction SBRT with real-time transperineal ultrasound guidance was feasible, with severe urinary and bowel AEs remaining uncommon, and associated with preserved clinically meaningful QoL, and encouraging preliminary biochemical control in patients with localized prostate cancer across all risk groups. This regimen may represent a practical intermediate strategy between conventional five-fraction SBRT and ultra-short fractionation, warranting longer follow-up and comparative prospective evaluation.

## Simple Summary

We prospectively evaluated a three-fraction SBRT schedule using real-time transperineal ultrasound guidance in patients with localized prostate cancer. Despite the predominantly elderly population, this approach was safe, well tolerated, and associated with low rates of severe urinary and bowel side effects, while maintaining patients’ quality of life. Early biochemical outcomes were encouraging, with a progressive decline in PSA levels and few biochemical failures during follow-up. These findings suggest that a three-fraction SBRT regimen may provide a practical balance between treatment convenience and clinical effectiveness, although longer follow-up is needed to confirm long-term outcomes.

## 1. Introduction

Prostate cancer is a major cause of morbidity and mortality worldwide, and the numbers are predicted to double by 2040 [1]. Because prostate cancer predominantly affects elderly men [2,3], many patients present with significant comorbidities that may limit surgical eligibility, making radiotherapy (RT) an increasingly important treatment option. External beam RT is a well-established curative treatment for localized prostate cancer across all risk groups [4,5]. In patients with intermediate- and high-risk disease, the addition of androgen deprivation therapy (ADT) improves oncological outcomes, with the duration of ADT tailored according to disease risk and longer courses recommended for patients with high-risk disease [6,7,8]. Continuous technological advances in treatment planning, image guidance, and delivery have progressively improved the therapeutic ratio of RT by enabling greater dose conformity while minimizing exposure of surrounding organs at risk (OARs). The development of hypofractionation has been strongly supported by radiobiological evidence. Prostate cancer is characterized by a remarkably low α/β ratio, estimated at approximately 1.5 Gy, which is lower than that of the adjacent normal tissues. This unique characteristic provides a strong biological rationale for delivering larger doses per fraction, potentially increasing tumour control while maintaining acceptable AE profiles [9,10]. Consequently, treatment schedules have progressively evolved from conventional fractionation to moderate hypofractionation and, more recently, to stereotactic body radiotherapy (SBRT). Randomized trials, including HYPO-RT-PC and PACE-B, have demonstrated that seven- or five-fraction SBRT provides biochemical control and side effect profiles comparable to those achieved with conventional or moderately hypofractionated RT, establishing SBRT as a standard treatment option for localized prostate cancer [11,12]. Building on these results, there is growing interest in further reducing the number of treatment fractions to improve patient convenience, optimize healthcare resources, and increase treatment accessibility without compromising safety or efficacy. This approach may be particularly advantageous for elderly patients, those living far from treatment centres, and individuals with significant medical comorbidities. As dose per fraction increases, however, treatment accuracy becomes progressively more critical. The prostate is subject to continuous intrafraction motion resulting from rectal filling, bladder filling, and patient movement. Even small positional changes may compromise target coverage or increase irradiation of adjacent OARs. For this reason, advanced image-guided radiotherapy techniques capable of real-time motion monitoring have become increasingly important in contemporary prostate SBRT. Several studies have demonstrated that continuous intrafraction monitoring allows margin reduction while maintaining adequate target coverage, thereby potentially improving treatment safety and reducing treatment-related AEs [13,14] and culminating in the delivery of single-fraction SBRT in carefully selected patients [15]. Taken together, these studies have highlighted the importance of precise target localization and continuous monitoring as enabling technologies for safe dose escalation and extreme hypofractionation. Several technologies are currently available for intrafraction prostate motion management, including MR-guided radiotherapy, electromagnetic transponder-based tracking, and real-time X-ray tracking of implanted fiducial markers. Although these approaches provide accurate target localization and motion monitoring, their implementation may require dedicated treatment platforms, additional infrastructure and costs, or invasive implantation procedures. Four-dimensional transperineal ultrasound (4D-TPUS) offers a non-invasive and non-ionizing alternative that enables continuous real-time prostate monitoring without implanted markers. Importantly, TPUS can be integrated with conventional linac-based treatment platforms, potentially improving accessibility and reducing procedural and technological burden compared with more resource-intensive tracking solutions. These characteristics provided the clinical and technical rationale for adopting 4D-TPUS in the present study, particularly in the context of an accelerated three-fraction regimen requiring accurate intrafraction motion management. Building on this stepwise clinical development, we hypothesized that three-fraction SBRT delivered with real-time four-dimensional transperineal ultrasound guidance could represent an effective compromise between established five-fraction regimens and emerging single-fraction approaches. Specifically, this study aimed to investigate whether treatment precision could be ensured while simplifying the procedural workflow by applying non-invasive real-time 4D-TPUS monitoring. The primary objective was to prospectively evaluate the safety of this approach, with the incidence of acute and late grade ≥ 2 genitourinary (GU) and gastrointestinal (GI) adverse events as the primary endpoint. Secondary objectives included the assessment of patient-reported quality of life (QoL), PSA kinetics, biochemical relapse-free survival (BRFS), and potential factors associated with treatment-related AEs.

## 2. Materials and Methods

This prospective single-centre study enrolled consecutive patients aged ≥ 18 years with histologically confirmed localized prostate cancer across all risk groups. Localized disease was defined as clinical stage < cT3b, N0, M0 based on PSMA PET/CT or conventional imaging when indicated according to disease risk. Patients with a previous transurethral resection of the prostate (TURP) or prostate enucleation were eligible. Key exclusion criteria included clinical stage ≥ cT3b, nodal or distant metastatic disease (N1/M1), prior pelvic radiotherapy, and active inflammatory bowel disease. The study was approved by the local ethics board. All enrolled patients were treated with a three-fraction SBRT regimen between November 2023 and October 2025.

All patients underwent a non-contrast-enhanced CT using the Clarity Autoscan system (Elekta AB, Stockholm, Sweden), which consists of a transperineal ultrasound (US) probe secured to the treatment couch via a dedicated leg support to facilitate patient positioning. Anatomical reproducibility was ensured by administering a rectal microenema and achieving a comfortably full bladder before both simulation and each treatment session. The clinical target volume (CTV) encompassed the entire prostate gland and a variable extent of the seminal vesicles based on clinical risk stratification and imaging findings. The PTV was generated by applying a 3 mm isotropic expansion to the CTV. OARs included the bladder, rectum, femoral heads, penile bulb, and small and large bowel. Treatment was delivered using volumetric modulated arc therapy (VMAT) on a VersaHD linear accelerator (Elekta AB) with one single 10 MV flattening filter-free (FFF) partial arc. SBRT was delivered every other day to a total dose of 30 Gy in three fractions whenever feasible; occasional prolongation of the overall treatment course was related to logistical and scheduling factors. Plans were optimized to ensure that the 95% isodose encompassed at least 95% of the PTV, while maintaining the 105% isodose below 0.1 cc of the PTV. As neither urethral catheterization nor MRI was used, accurate urethral delineation and tracking during treatment were not feasible. We therefore adopted an indirect urethral protection strategy aimed at achieving a homogeneous dose distribution and minimizing hotspots throughout the target volume, including the central gland where the urethra is typically located, a strategy associated with reduced acute and late GU AEs [16,17].

OAR constraints for the three-fraction regimen were derived from our institutional five-fraction SBRT constraints using a combination of linear–quadratic model-based conversion and pragmatic clinical adaptation. Assuming an α/β ratio of 3 Gy for late-responding normal tissues, the biologically effective dose was calculated as BED = *n*d [1 + d/(α/β)], where *n* is the number of fractions and d the dose per fraction. For the femoral heads and bowel bag, as well as for the dose constraints corresponding to 20% and 50% of the rectal volume and 40% of the bladder volume, the corresponding three-fraction doses were calculated by maintaining the same BED as the five-fraction constraints. The remaining rectal and bladder constraints were pragmatically adapted according to the prescription dose and the potential overlap between the PTV and the OARs. Specifically, the D 1 cc constraints for the rectum and bladder were set at 105% of the prescription dose, the dose constraint corresponding to 5% of the rectal volume was set at 100% of the prescription dose, and those corresponding to 10% of the rectal and bladder volumes were set at 90% of the prescription dose. The original five-fraction constraints, their three-fraction equivalents, and the corresponding BED/EQD2 values are reported in Table 1.

Image guidance was performed using CBCT (Elekta AB, Stockholm, Sweden) in combination with the Clarity real-time intrafraction monitoring [18]. A reference US image was acquired at simulation, registered to the planning CT to define the prostate volume for tracking, and verified against daily US and CBCT imaging before each fraction for real-time motion monitoring. Image quality was considered adequate for monitoring when the rectum, bladder, penile bulb, pubic symphysis, and prostate gland were clearly identifiable within the US field of view. A gating threshold of 2.5 mm displacement in any direction (antero-posterior, cranio-caudal, or left–right) was applied. If this threshold was exceeded for more than five seconds, beam delivery was automatically interrupted, and the shift was corrected either through automatic couch repositioning or by acquiring a new CBCT before resuming treatment.

ADT was prescribed according to the NCCN risk class, specifically directed to unfavourable intermediate and high risk. The choice of ADT agent was based on individual cardiovascular risk and treatment adherence.

The primary endpoint of the study was the incidence of acute and late grade ≥ 2 GU and GI AEs. Acute AEs were defined as those occurring within six months after treatment, whereas late AEs were defined as those occurring at or beyond six months. Secondary endpoints included changes in patient-reported QoL. Clinically meaningful changes in QoL were defined using the Minimal Clinically Important Difference (MCID), corresponding to a change of ≥0.5 standard deviations from baseline. Additional secondary endpoints included biochemical recurrence-free survival (BRFS), defined according to the Phoenix criteria (PSA nadir + 2 ng/mL), PSA kinetics, and the identification of factors associated with treatment-related AEs [19].

Clinical assessments were performed at baseline and during follow-up according to routine oncological follow-up schedules, typically every 3 months in the first year and every 6 months thereafter. Patient-reported outcomes were collected using the International Prostatic Symptom Score (IPSS), Expanded Prostate Cancer Index Composite for Clinical Practice (EPIC-CP) [20], and International Index of Erectile Function (IIEF-5), administered at baseline and during each follow-up visit. Questionnaire completion rates were calculated at each assessment time point as the proportion of expected questionnaires with evaluable data. Mixed-effects models included all available observations; however, no imputation of missing PRO data was performed. AEs were assessed during follow-up by treating physicians through structured interviews and graded according to the Common Terminology Criteria for Adverse Events (CTCAE) version 5.0, focusing on GU and GI AEs [21]. PSA levels were measured at baseline and during follow-up.

Motion analysis with the Clarity system was performed in the first 30 patients treated with the system and has been reported previously [18]. Following confirmation of the accuracy and feasibility of the workflow, additional patients were treated and included in the present study. The number of treatment interruptions and patient repositionings was retrospectively collected from the treatment and system log files for the full study population. A description of the 4D-TPUS-guided treatment workflow, including the main steps from simulation to treatment delivery and intrafraction motion management has been summarized in Supplementary Table S1.

Longitudinal changes in patient-reported outcomes were analyzed using linear mixed-effects models. Acute and late AEs were analyzed separately. Associations between population characteristics and grade ≥ 2 AEs were explored using univariable logistic regression models and reported as odds ratios (ORs) with 95% confidence intervals (CIs). Any predictor with a *p*-value < 0.1 in univariable analysis was considered for inclusion in the multivariable analysis, with a maximum of 4–5 predictors included for each outcome. Given the limited number of events for some AE endpoints, these multivariable models were intended primarily to explore associations among variables rather than to identify independent predictive factors. Intergroup differences were assessed using Fisher’s exact test for categorical variables and the Wilcoxon–Mann–Whitney test for continuous variables. BRFS was obtained by using a Kaplan–Meier method, with a confidence interval of 95%. No formal sample-size calculation or predefined feasibility criterion was specified; the study included consecutive eligible patients enrolled during the predefined study period. All analyses were performed using Stata software (version 13; StataCorp, College Station, TX, USA).

## 3. Results

### 3.1. Patient Characteristics

Seventy-two patients with a median age of 78 (56–87) years were enrolled. The median prostate volume was 64 cc (range, 20–140 cc). The median PSA level before RT was 7.9 ng/mL (range, 1.5–65.0 ng/mL), and the median baseline IPSS was 6 (range, 0–33). Further details on baseline patient characteristics are provided in Table 2.

Based on the ISUP GG classification, 58.4% of patients had GG 1–2 disease (15.3% and 43.1%, respectively), 25.0% had GG 3, and 16.6% had GG 4–5 disease. ADT was prescribed in thirty patients (41.7%), in accordance with current guidelines [4,5] and patient comorbidities. Among these patients, twenty-four received a luteinizing hormone-releasing hormone (LHRH) analogue, two were treated with a gonadotropin-releasing hormone (GnRH) antagonist, and four received bicalutamide 150 mg daily. The median duration of hormonal therapy was 11 months (range, 4–18 months). The median follow-up was 16 months (range, 6–27), the median time from diagnosis to RT was 5 months (range, 1–12) and the median total RT duration was 7 days (range, 4–21). The median interval from CT simulation to RT initiation was 32 days (range, 14–73) and the median CTV and PTV volumes were 66.7 cc (range, 36.6–140.4 cc) and 106.5 cc (range, 65.3–194.1 cc), respectively.

### 3.2. Adverse Events

Using the prespecified 6-month definition, acute grade ≥ 2 GU AEs occurred in 25/72 patients (34.7%), mainly consisting of dysuria and increased urinary frequency, whereas acute grade ≥ 2 GI AEs occurred in 6/72 patients (8.3%), primarily presenting as rectal tenesmus. Late grade ≥ 2 GU and GI AEs occurred in 13.9% and 4.2% of patients, respectively. Only one patient experienced acute grade 3 GU AEs (severe cystitis) and another one experienced late grade 3 GU AE (acute urinary retention). No grade 4–5 AEs were observed. The AEs rate is summarized in Table 3.

On univariate analysis, a larger CTV, baseline IPSS, diabetes, and previous pelvic surgery correlated with grade ≥ 2 GU acute AEs (respectively, OR = 1.03, 95% CI: 1.01–1.06, *p* = 0.017; OR = 1.1, 95% CI: 1.03–1.25, *p* = 0.007; OR = 5.6, 95% CI: 1.65–19.06, *p* = 0.006; OR = 8.4, 95% CI: 2.32–30.80, *p* = 0.001).

**Figure 1:**
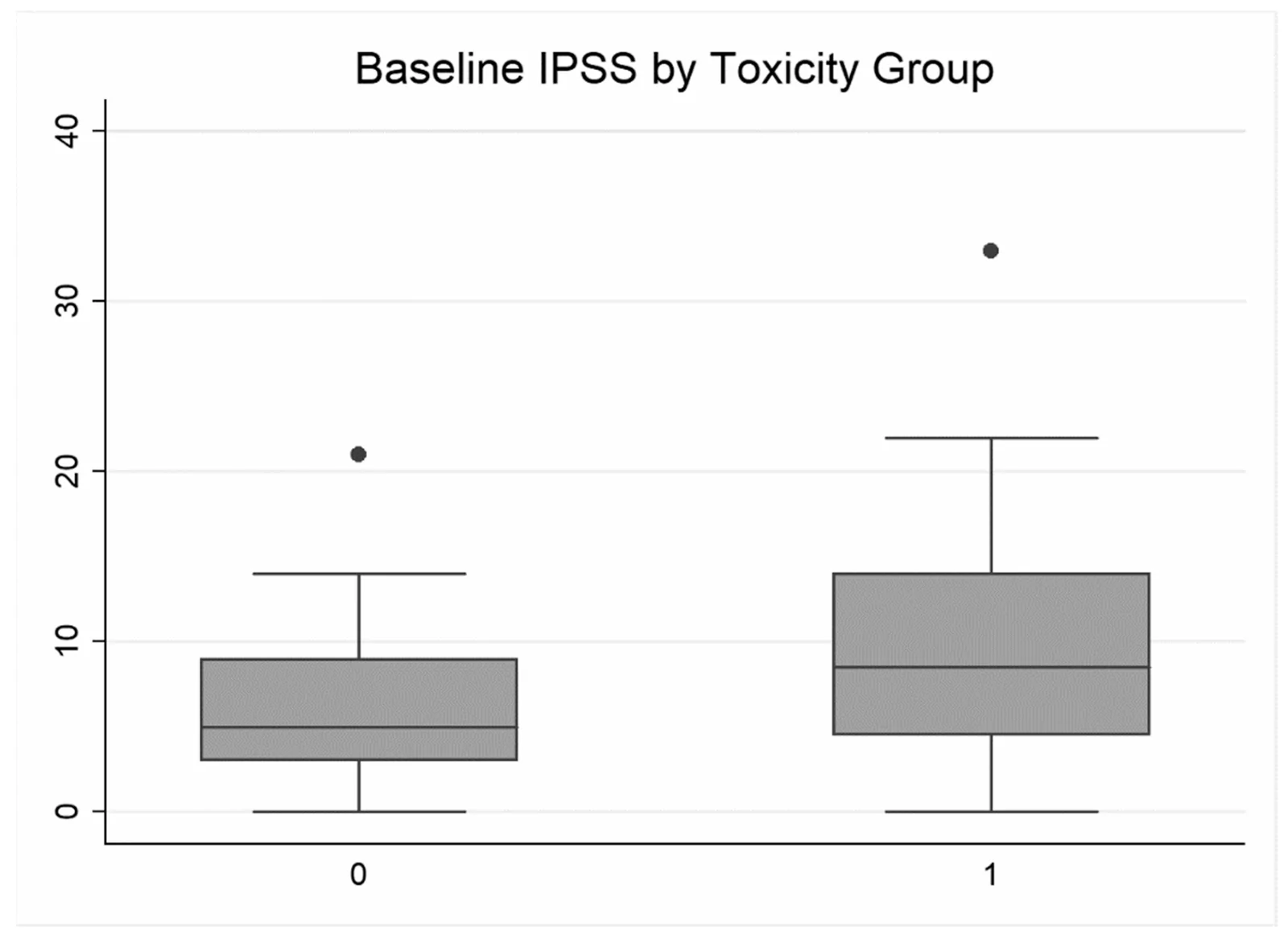
Box plot of baseline IPSS stratified by acute GU AEs.

On multivariate analysis, only higher baseline IPSS remained significantly associated with acute GU AEs (aOR = 1.12, 95% CI: 1.00–1.26; *p* = 0.045). Patients who experienced acute grade ≥ 2 GU events had a median IPSS of 9, while those who did not had a median of 5. Notably, the only patient who developed grade 3 acute GU AEs had a baseline IPSS of 13. This association is illustrated in Figure 1.

IPSS and smoking were correlated with acute grade ≥ 2 GI events at univariate analysis (respectively, OR = 1.26, 95% CI: 1.08–1.47, *p* = 0.003; OR = 8.43, 95% CI: 1.42–50.07, *p* = 0.019), while prior pelvic surgery emerged as the only significant factor associated with the development of late grade ≥ 2 GU AEs (OR = 8.83, 95% CI: 2.07–37.62, *p* = 0.003).

### 3.3. Patient-Reported Outcomes (QoL)

In a mixed-effects model accounting for repeated measures, patient-reported outcomes demonstrated heterogeneous temporal patterns following treatment, as shown in Table 4. Bowel function remained stable throughout follow-up, whereas hormone-related symptoms showed an early deterioration. Urinary symptoms, including IPSS, obstructive symptoms, and incontinence, worsened predominantly at late follow-up. Sexual function declined early after treatment and remained persistently impaired over time. Consistent with these findings, overall EPIC-CP scores showed a sustained deterioration during follow-up. Considering the three main time points used for the analyses—baseline, acute, and late—the questionnaire completion rates were 98.6%, 90.3%, and 55.6%, respectively, for the IPSS; 91.7%, 86.1%, and 55.6% for the EPIC-CP; and 76.4%, 62.5%, and 37.5% for the IIEF-5. Although several longitudinal changes from baseline were statistically significant, the estimated mean changes at the group level remained below the corresponding MCID thresholds, indicating that they were not clinically meaningful on average. However, when patient-reported outcomes were evaluated at the individual patient level, clinically meaningful deteriorations were observed in a proportion of patients, as reported in Table 5.

### 3.4. Biochemical Outcomes

A significant reduction in PSA levels was observed over time, decreasing from a median of 7.90 ng/mL (range 1.50–65.00) at baseline to 0.44 ng/mL (range 0.01–26.00) at last follow-up.

**Figure 2:**
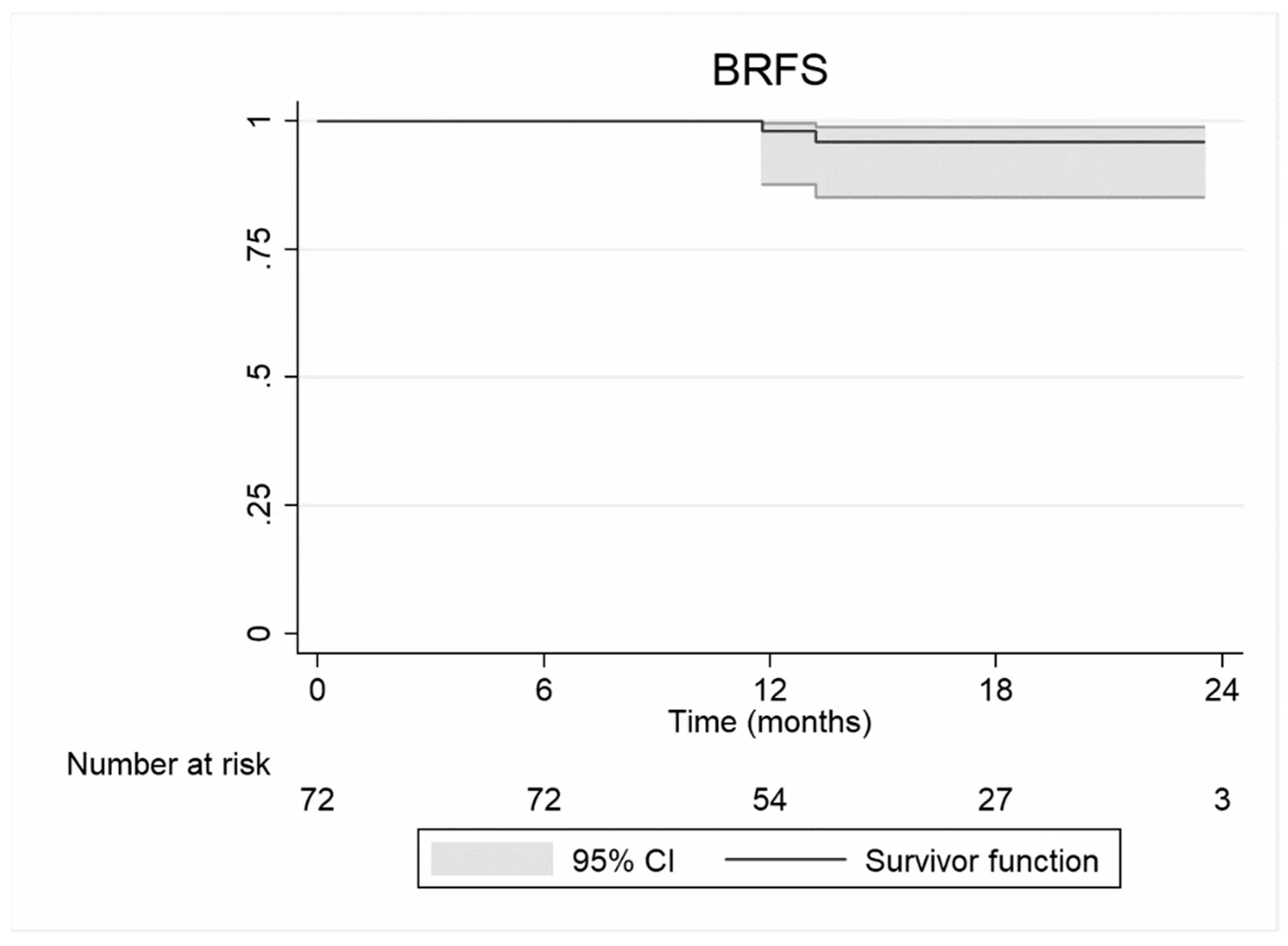
Kaplan–Meier curve of BRFS.

The Kaplan–Meier survival analysis demonstrated an estimated BRFS of 98.2% at 12 months (95% CI: 87.8–99.7%) and 96.1% at 24 months (95% CI: 85.2–99.0%), as shown in Figure 2. Two patients developed biochemical recurrence, were further evaluated with PSMA PET with detection of distant metastasis.

### 3.5. Intrafraction Motion Analysis

Detailed analysis of intrafraction motion with the Clarity system has been reported elsewhere [18]. Briefly, the predominant direction of motion was posterior, followed by inferior displacement. The magnitude of intrafraction motion remained generally limited over the monitored treatment time (median monitoring duration, 6.4 min). The proportion of fractions with a prostate displacement of ≥3 mm in any of the three spatial directions increased with treatment time (i.e., time from CBCT acquisition to beam off), rising from >5% at 3.8 min to 11.1% and 17.4% at 5 and 6 min, respectively. These findings highlight the importance of real-time motion monitoring when using a 3 mm PTV margin. In the present study, among 216 monitored treatment fractions for the 72 patients, 174 fractions (81%) were delivered without intrafraction motion exceeding 2.5 mm, whereas 42 fractions (19%) experienced motion beyond this threshold. Overall, 29 patients (40%) experienced at least one treatment interruption, accounting for 77 interruptions across the study population. Of these, 37 were transient interruptions, during which the prostate spontaneously returned within the predefined tolerance without the need for patient repositioning, whereas 40 interruptions required patient repositioning.

## 4. Discussion

The present prospective study demonstrates that three-fraction SBRT delivered with real-time transperineal ultrasound guidance is feasible in patients with localized prostate cancer across all risk groups. Despite including an elderly population (median age 78 years) and a substantial proportion of unfavourable intermediate- and high-risk disease (44.4%), treatment was associated with a low incidence of severe AEs, limited clinically meaningful deterioration in patient-reported quality of life, and encouraging preliminary biochemical control. These findings support three-fraction SBRT as a practical strategy for safely reducing treatment duration while maintaining favourable early clinical outcomes. Randomized trials such as HYPO-RT-PC and PACE-B [11,12] have established ultra-hypofractionated RT as a standard treatment option, and most of the clinical experience has been generated using five- or seven-fraction schedules [22]. More recently, technological advances have stimulated interest in further reducing the number of fractions to improve patient convenience, increase treatment capacity, and reduce healthcare costs. In this context, three-fraction SBRT may represent a logical intermediate step between established five-fraction regimens and the more experimental one- and two-fraction approaches [23]. Delivering 10 Gy per fraction preserves many of the logistical advantages of extreme hypofractionation while retaining the potential radiobiological and dosimetric benefits of dose fractionation. The OAR constraints were derived from our institutional five-fraction SBRT constraints using the LQ model with an α/β ratio of 3 Gy for late-responding normal tissues. Although this provided a pragmatic framework for biological dose conversion, the validity of the LQ model at high doses per fraction remains uncertain. Therefore, these constraints should be regarded as approximate biological equivalents rather than formally validated thresholds for three-fraction SBRT. To our knowledge, there is only one published prospective trial evaluating three-fraction prostate SBRT, and it reported acceptable rates of acute and late GU and GI AEs [24]. However, these results were obtained using a highly complex workflow incorporating fiducial markers, rectal spacers, bladder catheterization, and multiparametric MRI. By contrast, our protocol relied exclusively on CBCT and continuous transperineal ultrasound tracking without invasive localization procedures. This simplified workflow may facilitate broader implementation while maintaining precise intrafraction motion management and acceptable side effects. Such an approach may be particularly attractive for centres without access to MRI-guided radiotherapy or robotic tracking systems. The intrafraction motion analysis by Panizza et al. [18] supports the role of real-time monitoring in this setting. The increasing proportion of fractions exceeding a displacement of 3 mm with longer treatment times suggests that clinically relevant motion may occur during treatment, including within the typical treatment duration of our cohort. Thus, combining a 3 mm PTV margin with real-time monitoring and beam interruption for motion exceeding the predefined threshold enabled the detection and correction of out-of-tolerance displacements while minimizing unnecessary treatment interruptions. In the aforementioned analysis [18], smaller motion thresholds/margins were associated with substantially more frequent interruptions, whereas larger margins reduced the proportion of fractions requiring intervention to 1.0–2.3% at the corresponding time points of 5 and 6 min, potentially reducing the added value of real-time monitoring.

Acute grade ≥ 2 GU AEs occurred in 34.7% of patients, a non-negligible rate that appears higher than that reported in several contemporary five-fraction SBRT series and in the available prospective three-fraction experience. However, several factors should be considered when interpreting these findings. First, our cohort included patients across the entire spectrum of disease risk, without excluding individuals based on prostate volume, baseline urinary symptoms (as assessed by IPSS), or a history of TURP or other prostate surgery, thereby reflecting routine clinical practice rather than highly selected trial populations. Specifically, baseline urinary function emerged as an important determinant of side effects. Patients developing acute GU AEs grade ≥ 2 had significantly higher baseline IPSS values, confirming previous observations that pre-existing lower urinary tract symptoms represent one of the strongest predictors of urinary morbidity after prostate SBRT [25,26]. What emerged emphasizes the importance of careful patient selection and pre-treatment counselling when considering accelerated treatment schedules, particularly in patients with significant baseline urinary dysfunction. Second, no direct urethral sparing was applied. Although hotspots within the PTV were strictly limited, the absence of urethra-specific dose constraints should be considered when interpreting the observed rate of acute GU AEs. Third, acute AEs were prospectively collected over six months following treatment, whereas most SBRT studies define acute events within 90 days, potentially increasing the reported incidence of treatment-related symptoms.

Importantly, severe events remained uncommon, with only one acute and one late GU grade 3 AEs, and no grade 4 or 5 AEs. Gastrointestinal AEs remained particularly low throughout follow-up, suggesting that the combination of small PTV margins and continuous intrafraction monitoring effectively limited unnecessary rectal irradiation. Patient-reported outcomes further support the tolerability of this regimen. Although statistically significant changes were observed in urinary, sexual, and hormonal domains, none exceeded the predefined threshold for MCID. This distinction between statistical significance and clinical relevance is particularly important in elderly patients, in whom baseline urinary and sexual dysfunction are common and treatment-related changes should be interpreted within the broader clinical context. The stability of bowel-related QoL is consistent with the low incidence of gastrointestinal AEs observed clinically.

The interpretation of PSA kinetics and patient-reported outcomes should also account for the potential confounding effect of ADT, which was administered to a relevant proportion of patients. In particular, ADT may have contributed to the observed PSA decline and may independently affect hormonal symptoms and sexual function, including IIEF-5 scores. Therefore, biochemical and QoL outcomes should not be attributed exclusively to the radiotherapy regimen. Longer follow-up will be required to determine whether these encouraging early outcomes translate into durable disease control comparable with established five-fraction schedules.

Finally, our results should be viewed within the broader evolution of prostate SBRT. While single-fraction treatments such as ABRUPT, PROSINT, PROFAST and ONE SHOT [15,27,28,29] and emerging two-fraction protocols [30,31] continue to push the boundaries of hypofractionation, these approaches rely on highly specialized technology and invasive preparation. Three-fraction SBRT occupies an intermediate position, combining a substantial reduction in treatment burden with a workflow based on technologies that are increasingly available in modern radiotherapy departments. In this regard, real-time transperineal ultrasound guidance may represent an effective, non-invasive, and potentially cost-efficient solution for intrafraction motion management.

Several limitations of the present study should be acknowledged: it represents the experience of a single institution with a relatively limited number of patients and a median follow-up of only 16 months, precluding definitive conclusions regarding long-term AE profile and oncological efficacy. In particular, although 24-month BRFS was estimated at 96.1%, only three patients remained at risk; therefore, oncological outcomes should be interpreted with caution and considered preliminary, pending confirmation with longer follow-up and larger cohorts. Furthermore, the heterogeneous inclusion of all risk groups and the use of ADT according to clinical indications introduce additional variability. Conversely, the prospective design, systematic collection of patient-reported outcomes, and standardized ultrasound-based motion monitoring strengthen the clinical relevance of our findings.

## 5. Conclusions

Three-fraction SBRT with real-time transperineal ultrasound guidance was feasible and well tolerated in patients with localized prostate cancer across all risk groups. Despite the inclusion of elderly population, treatment was associated with a low incidence of severe AEs, preservation of clinically meaningful QoL, and encouraging preliminary biochemical control. By combining a substantially shortened treatment course with a non-invasive and widely applicable image-guidance strategy, this approach may bridge the gap between established five-fraction SBRT and emerging ultra-extreme hypofractionation. Confirmation through longer follow-up and randomized comparisons will be essential before broader adoption.

## Figures and Tables

**Table 1:** Dose–volume constraints for OARs used in the SBRT treatment plans delivered with 10 Gy × 3 fractions. The three-fraction constraints were derived from the institutional constraints for the 5-fraction regimen using a combination of linear–quadratic model-based conversion and pragmatic clinical adaptation, assuming an α/β ratio of 3 Gy for late-responding normal tissues [17].

OARs	Constraints 10 Gy × 3 fx	Constraints 7.25 Gy × 5 fx
	EQD2_3_ 78.0 Gy	EQD2_3_ 74.3 Gy
Femoral heads	V 12.1 Gy < 5%	V 14.5 Gy < 5%
Rectum	V 30.0 Gy < 5% V 27.0 Gy < 10% V 23.5 Gy < 20% V 15.0 Gy < 50% D 1 cc < 31.5 Gy	V 36.25 Gy < 5% V 32.625 Gy < 10% V 29.0 Gy < 20% V 18.1 Gy < 50% D 1 cc < 38.1 Gy
Bladder	V 27.0 Gy < 10% V 15.0 Gy < 40% D 1 cc < 31.5 Gy	V 32.625 Gy < 10% V 18.1 Gy < 40% D 1 cc < 38.1 Gy
Bowel PRV	D 0.035 cc < 28.2 Gy	D 0.035 cc < 35 Gy

**Table 2:** Baseline patient characteristics presented as *n* (%). Overall cohort: *n* = 72.

**Variable**	***n* (%)**
Diabetes	15 (20.8%)
Smoking	10 (13.9%)
Hypertension	52 (72.2%)
Anticoagulant use	15 (20.8%)
Statin use	35 (48.6%)
Alpha-blocker use	56 (77.8%)
Coronary artery disease	20 (27.8%)
Pelvic surgery	15 (20.8%)
Prior TURP	9 (12.5%)
**ISUP Grade group**	***n* (%)**
1	11 (15.3%)
2	31 (43.1%)
3	18 (25.0%)
4	11 (15.3%)
5	1 (1.3%)
**Clinical T stage**	***n* (%)**
cT1c	12 (16.6%)
cT2a	13 (18.1%)
cT2b	16 (22.2%)
cT2c	27 (37.5%)
cT3a	4 (5.6%)
**NCCN risk group**	***n* (%)**
Low risk	2 (2.8%)
Favourable intermediate risk	38 (52.8%)
Unfavourable intermediate risk	14 (19.4%)
High risk	18 (25.0%)

**Table 3:** AEs profile.

AEs	G0	G1	G2	G3	G4–5
Acute GU	31 (43%)	16 (22.2%)	24 (33.3%)	1 (1.4%)	0
Acute GI	56 (77.8%)	10 (13.9%)	6 (8.3%)	0	0
Late GU	48 (66.7%)	14 (19.4%)	9 (12.5%)	1 (1.4%)	0
Late GI	67 (93%)	2 (2.8%)	3 (4.2%)	0	0

**Table 4:** Longitudinal changes in patient-reported outcomes and the corresponding *p*-value from the mixed-effects model.

Questionnaire Domain	Baseline	Acute	Late	*p*-Value
IPSS	6 (0–33)	5 (0–18)	8 (0–24)	**0.006**
QoL	9 (0–27)	12 (0–27)	14 (0–29)	**<0.001**
Incontinence	0 (0–6)	0 (0–6)	1 (0–7)	**0.001**
Obstruction	2 (0–11)	2 (0–12)	3 (0–9)	**<0.001**
Bowel	0 (0–4)	0 (0–9)	0 (0–7)	NS
Sex	5 (0–12)	8 (0–12)	8 (0–12)	**0.006**
Hormone	0 (0–10)	2 (0–10)	1 (0–7)	**0.002**
IIEF-5	13 (4–25)	7 (5–25)	5 (5–25)	**0.027**

**Table 5:** Proportion of patients who experienced clinically meaningful deterioration for each domain.

Questionnaire Domain	Acute	Late
IPSS	2/65 (3%)	10/40 (25%)
QoL	15/62 (24%)	10/40 (25%)
Incontinence	6/60 (10%)	9/40 (23%)
Obstruction	18/60 (30%)	14/40 (35%)
Bowel	8/60 (13%)	4/40 (10%)
Sex	17/59 (29%)	6/39 (15%)
Hormone	20/59 (34%)	4/39 (10%)
IIEF-5	9/45 (20%)	3/27 (11%)

## Data Availability

Research data are stored in an institutional repository and can be shared upon reasonable request to the corresponding author.

## Supplementary Materials

The following supporting information can be downloaded at https://www.mdpi.com/article/10.3390/cancers18182954/s1, Supplementary Table S1: Practical Workflow for 4D-TPUS-Guided Three-Fraction Prostate SBRT.
