# Supplementary material for: Prospective Evaluation of Three-Fraction SBRT with 4D Transperineal Ultrasound Intrafraction Motion Monitoring for Localized Prostate Cancer
Source: Cancers (Basel). 2026 Sep 13;18(18):2954. doi: 10.3390/cancers18182954 (PMC13604539; doi:10.3390/cancers18182954)
Supplement: Supplementary file 1 [file cancers-18-02954-s001.zip › cancers-4513117-supplementary.pdf]

Supplementary Table S1: "Practical Workflow for 4D-TPUS-Guided Three-Fraction Prostate SBRT."

Step	Procedure	Key technical details
**1**	Patient preparation	Microenema; comfortably full bladder
**2**	Simulation	CT with TPUS probe positioned and secured
**3**	TPUS reference acquisition	Reference US dataset acquired at simulation
**4**	Planning	CTV definition; 3-mm isotropic PTV; OAR constraints
**5**	Pre-treatment setup	Reproduction of bladder/rectal preparation and TPUS probe position
**6**	Image guidance	CBCT-based localization/correction
**7**	Intrafraction monitoring	Continuous 4D-TPUS; 2.5-mm threshold in AP/CC/LR
**8**	Gating/correction	Beam interruption if >2.5 mm persists >5 s; couch correction or repeat CBCT before resuming
